# Saudi guidelines for testing and treatment of latent tuberculosis infection

**DOI:** 10.4103/0256-4947.59373

**Published:** 2010

**Authors:** Hamdan H. Al Jahdali, Salim Baharoon, Abdullah A. Abba, Ziad A. Memish, Abdulrahman A. Alrajhi, Ali AlBarrak, Qais A. Haddad, Mohammad Al Hajjaj, Madhukar Pai, Dick Menzies

**Affiliations:** a From the Department of Medicine, King Saud University for Health Sciences, King Abdulaziz Medical City, Riyadh; b From the King Khalid University Hospital and College of Medicine, King Saud University, Riyadh; c From the King Faisal Specialist Hospital and Research Centre, Riyadh; d From the Riyadh Armed Forces Hospital, Riyadh; e From the Security Forces Hospital, Riyadh; f From the Montreal Chest Institute & Department of Epidemiology & Biostatistics, McGill University, Montreal, Canada

## Abstract

Pulmonary tuberculosis is a common disease in Saudi Arabia. As most cases of tuberculosis are due to reactivation of latent infection, identification of individuals with latent tuberculosis infection (LTBI) who are at increased risk of progression to active disease, is a key element of tuberculosis control programs. Whereas general screening of individuals for LTBI is not cost-effective, targeted testing of individuals at high risk of disease progression is the right approach. Treatment of those patients with LTBI can diminish the risk of progression to active tuberculosis disease in the majority of treated patients. This statement is the first Saudi guideline for testing and treatment of LTBI and is a result of the cooperative efforts of four local Saudi scientific societies. This Guideline is intended to provide physicians and allied health workers in Saudi Arabia with the standard of care for testing and treatment of LTBI.

Over the last century, the tuberculin skin test (TST) has been used extensively in epidemiological surveys and as a clinical test for the diagnosis of latent *Mycobacterium tuberculosis (MTB)* infection (LTBI) in different populations throughout the world.^1^–^4^ It is mainly used for the identification and treatment of persons infected by *MTB,* who are at high risk of progression to active disease.^4^ This strategy has proved to be effective in tuberculosis (TB) control because preventive treatment of latently infected people diminishes the risk of subsequent development of active TB by about 90%.^4^–^7^

The TST uses a relatively crude mix of antigens from MTB. As a result, false-positive reactions can occur because of previous Bacillus Calmette-Guérin (BCG) vaccination or sensitization to nontuberculous mycobacteria (NTM). This complicates the interpretation of the TST as tuberculin reactions caused by BCG or NTM cannot be distinguished with absolute certainty from those caused by latent TB infection. However, the wealth of epidemiological information has allowed the formulation of recommendations for informed interpretation of the TST in most clinical situations.^4^,^8^ We believe, however, that many practitioners remain reluctant to use the TST, and therefore, do not give therapy for latent TB infection even to patients at high risk of reactivation.

This statement provides the first set of national recommendations for targeted tuberculin testing and treatment regimens for persons with latent tuberculosis infection. We strongly believe that targeted testing and treatment of individuals with latent tuberculosis infection, who are at increased risk of progression to active disease, is a key component of tuberculosis control. Infected persons who are considered to be at high risk for developing active TB should be offered treatment of LTBI, unless there are absolute contraindications for therapy.

This statement by the Saudi Thoracic Society (STS), the Saudi Society of Medical Microbiology and Infectious Disease (SSMMID), and Saudi Association of Public Health (SAPH) and Society of Family and Community Medicine, provides the first national recommendation for targeted tuberculin testing and treatment regimens for person with latent tuberculosis infection in Saudi Arabia.

## Prevalence of LTBI in Saudi Arabia

The total number of reported tuberculosis cases in the year 2008 was 3 918 cases in a population of approximately 24 807 273. TB not Tuberculosis in Saudi Arabia affects mainly young and middle-aged individuals; most of them aged between 15 and 44 years old. The incidence of all cases was estimated at 15.8/100 000; the incidence of smear-positive tuberculosis was 8.2/100 000. However, WHO 2007 estimation of the incidence of TB new cases was 46/100 000 population/year, the incidence of new smear positive cases was 21/100 000 population/year and the estimated prevalence of all forms of TB cases was 65/100 000 population/year.^10^ Al Kassimi et al in 1993^9^ conducted the first comprehensive and nationwide tuberculin survey in the Saudi Arabian general population with urban/rural stratification. Using a definition of a positive tuberculin test of 10 mm or more, 33% of the subjects had a positive TST, and 56% were aged 45 years and older. Given that false positive reactions can occur in almost all populations, the most important determinant of the utility of a TST is the expected prevalence of true latent TB infection. The incidence of smear-positive cases of pulmonary tuberculosis varies widely between countries. Based on the observation by Styblo^9^ of the relationship between the incidence of smear-positive pulmonary TB and the annual risk of TB infection (ARI), one can calculate the ARI based on the expected prevalence of latent TB infection in persons of a given age. In Saudi Arabia, the estimated incidence of active TB (all forms) is 46 per 100 000 and the incidence of smear-positive TB is 21 cases per 100 000, which gives an annual risk of infection of 0.35%.^10^ Using the Stabylo^9^ calculation, the prevalence of LTBI at 10 and 20 years is 3.4% and 6.7% respectively, placing Saudi Arabia in the intermediate prevalence (2%-14%) category.^11^

## Administrative technique for the TST

The administration of TST requires proper technique by trained professionals. A standard tuberculin syringe with a 27-gauge needle should be used to inject five tuberculin units of purified protein derivative (PPD) or two tuberculin units of RT-23 intradermally. Usually, the volar or inner surface of the forearm is used. The area of the skin selected for testing should be free of any cutaneous or subcutaneous lesion that could interfere with any interpretation. The injection should be intradermal, and neither too deep nor too shallow. A small wheal measuring approximately 5 mm in diameter should be elicited at the time of injection. If incorrect administration is suspected, a second test dose can be given immediately at a site that is at least 5 cm away from the first one.^4^ As a result of its tendency to be adsorbed by glass and plastics, tuberculin materials should never be transferred to secondary containers. It should be administered as soon as feasible after filling the syringe, ideally within 20 minutes. Long-term storage of prefilled syringes is not recommended. Tuberculin material should be kept refrigerated but not frozen, and most importantly, kept protected from light.

## Reading the TST

The tuberculin reaction is a classic, delayed type, hypersensitivity immune reaction. This means that the reaction begins within 24 hours, is maximal between 48 and 72 hours after injection, and then wanes over the next few days. Reading is done between 48 and 72 hours after administration.

The transverse diameter of the induration, but not erythema, is demarcated using the ball-point technique. The induration is measured and recorded in millimeters. If there is no reaction, the size should be recorded as 0 mm and not simply as “negative”. Similarly readings of “doubtful” or “positive” are discouraged, particularly in individuals in whom repeated testing is done (see below).^4^

Adverse reactions to tuberculin skin testing are uncommon. Local allergic reactions to tuberculin or its components can occur in 2% to 3% of those tested usually in the form of a rash on the arm. Generalized rash has also been observed. These reactions begin shortly after injection and disappear within 24 hours. Severe blistering is possible, for which topical steroids are frequently given, although there is no evidence of their efficacy. The blistered area should be covered with a dry dressing and the patient advised to keep it clean and not to scratch it.^4^

## Interpretation of the TST

### False-negative TST

Many factors can cause false-negative tests including viral, bacterial, or fungal infections. The most important cause of a false-negative TST is HIV infection. In HIV-infected persons, the likelihood of a false-negative TST is inversely proportional to the CD4 count: it is uncommon at CD4 counts above 500/mL, and almost universal when the CD4 count is less than 200/mL. Saudi Arabia has a low prevalence of HIV infection of less than 0.01%.^12^,^13^ HIV infection is therefore not an important cause of a false-negative TST in Saudi Arabia.

It is important to note that patients with active TB can have false-negative tests at the time of diagnosis, particularly those with more advanced disease.^4^ Anergy testing with control antigens to which most normal adolescents and adults should react, such as mumps and *Candida*, have been used to determine whether people can have true- or false-negative tuberculin reactions. However, anergy testing is not routinely recommended in persons who are HIV-infected or otherwise immunocompromised because of the evidence that this testing is unreliable.^4^ Other causes of false-negative TSTs are presented in Table 1.

**Table 1:** Causes of false-negative tuberculin skin testing

Technical factors: (potentially correctable)
- Defective antigens because of improper storage (exposure to light or heat) or manufacturing
- Contamination, improper dilution
- Injection of too little tuberculin, or too superficial, or too deeply (should be intradermal)
- Prolonged storage within the syringe before administration (more than 20 minutes)
- Inexperienced or biased reader
- Error in recording
Biological factors: (not correctable)
- Viral, bacterial, or fungal infections
- HIV (especially if CD4 count <200)
- Live virus vaccination within the past two months
- Metabolic derangement, protein depletion, chronic renal failure, severe malnutrition, stress (surgery, burns)
- Concurrent use of immunosuppressive drugs: (corticosteroids, TNF inhibitors, and others)
- Very young <6 months or elder
- Diseases of lymphoid organs: (lymphoma, chronic lymphocytic leukemia, sarcoidosis)

### False-positive TST

BCG vaccination was started in 1964 in Saudi Arabia and a 95.9% coverage rate was achieved by 2007.^14^ The BCG vaccine is given at birth without any booster doses. A case-control study in Saudi Arabia determined the protective effect of the BCG vaccine using the same BCG strain (freeze-dried glutamate BCG, Japan laboratory Tokyo).^15^ This study demonstrated an 82% protective effect in the 5-14-year-old age group, but no protective effect 25 years after vaccination.^15^

In several other studies following BCG vaccination in infancy, tuberculin reactivity was seen to wane rapidly so that there was little discernible effect by the age of five years. However, those vaccinated at a later age have larger TST reactions that wane more slowly, with 15% to 25% of the reactions persisting beyond ten years.^16^–^22^

A meta-analysis including more than 24 studies found that only 1% of patients who received BCG during infancy were TST positive if tested more than ten years after BCG vaccination. However, there was a greater and more long-lasting effect on TST if BCG was given later in life, such as during the primary or secondary school-going years.^23^ Most of the international guidelines recommend ignoring BCG's effect on the interpretation of TST in persons at increased risk of developing active TB.^4^

## Nontuberculous mycobacteria

Nontuberculous mycobacteria (NTM) are another major cause of false-positive TST.^4^,^20^,^24^ The prevalence of NTM varies considerably between regions, countries, and even within countries. There are few studies of the prevalence of NTM in the Middle East. Two recent hospital-based studies in Kuwait and Saudi Arabia identified NTM in 18 of 325 (5.9%) and 70 of 286 (24.5%) specimens, respectively.^25^,^26^ An earlier similar study from Saudi Arabia gave an intermediate figure of 9%.^27^ A nationwide population-based survey, however, revealed a much lower figure of 0.004%.^9^ A recent meta-analysis involving more than 18 studies found that NTM is not a clinically important cause of false-positive TST, particularly in areas of high TB prevalence.^28^

## Serial tuberculin testing (conversion or boosting)

When repeated tuberculin testing has been performed, a substantial number of individuals may manifest an increased tuberculin reaction on their second test in the absence of any obvious exposure. It is now realized that this reflects an anamnestic response representing the restimulation of previously acquired immunity from earlier exposure.^29^ This phenomenon, termed “boosting”, results from a previous mycobacterial exposure, including remote BCG vaccination, nontuberculous mycobacterial exposure, or remote infection with MTB.^4^,^29^

It is important to distinguish boosting from the phenomenon of tuberculin conversion which occurs after an initial tuberculin infection. This is because the risk of developing active tuberculosis in persons who manifest the “boosting” phenomenon is actually lower than those with an initial positive TST, and much lower than in persons with tuberculin conversion.^29^ In persons with conversion, the risk of disease is between 5% and 20% over the next two years alone.

It is difficult to distinguish the phenomena of boosting and conversion simply on the basis of size, although if the second reaction is >15 mm, it is most likely to be conversion. If the second TST is performed soon (within two weeks) after the first and there is no intervening exposure, then boosting is most likely the cause for a positive TST. If the second test is performed months to years after the first, and there has been exposure to MTB (such as contacts of active cases), then the cause for a positive TST is considered to be recent TST conversion, indicating TB infection.^29^

In some clinical situations, it may be necessary to repeat the TST on a periodic basis. An initial two-step testing protocol is recommended in this situation. The most common situation is when persons enter an environment where there might be a risk of exposure to TB (e.g., persons entering professions or facilities with an increased risk of occupational exposure such as health care or prisons).^4^,^29^

If the initial TST is negative (<10 mm), then a second test should be performed 1 to 4 weeks later to elicit the booster phenomenon.^4^ Boosting can be seen after intervals as long as two years, but this phenomenon is maximal if the two tests are between one and four weeks apart, and a shorter interval is always more practical. If the second tuberculin test is negative (<10 mm), the individual can be considered to be truly negative. If on subsequent retesting, the tuberculin test is positive, this can be considered as a true conversion. This is strong evidence of new infection and should therefore be treated.^29^

A TST conversion is defined as a TST reaction of 10 mm or more plus as an increase of 6 mm or more of induration from the previous TST results within a two-year period, regardless of age.^4^,^29^,^30^ The ATS/CDC/IDSA definition of conversion requires an increase of 10 mm or more from the previous TST result.^4^,^7^ This more stringent criteria will be more specific but less sensitive to detect conversion.

## Indications for TST and who should be treated

Routine screening of asymptomatic subjects is not recommended.^4^,^31^ The risk-benefit ratio for treating LTBI depends principally on whether the individual is truly infected and is at substantial risk of developing active TB disease. Therefore, TST screening for LTBI should be reserved for those groups with a high risk of recent infection, or who are at high risk of progression from LTBI to TB disease as outlined in Tables 2 and 3.

**Table 2:** Indications for tuberculin skin testing.

A. Individuals with increased risk for new infection: (all patients should be tested regardless of age)
- Close contacts of persons with active pulmonary TB
- Persons whose tuberculin skin tests have converted to positive (≥10 mm induration and >6 mm increase) within the past two years
- Persons who live or work in clinical or institutional settings where TB exposure may be likely (e.g., hospitals, prisons, nursing homes, microbiology labs)
B. Individuals with clinical conditions associated with increased risk of reactivation:
1. High risk (all patients should be tested regardless of age)
- Persons with HIV infection
- Persons who never received antituberculosis therapy with abnormal chest x-ray with apical fibronodular changes typical of healed TB (not including granuloma)
- Persons with certain medical conditions (e.g., silicosis, chronic renal failure on dialysis
- Transplant, lymphoma, leukemia chemotherapy
2. Moderate risk (only patients less than 65 years of age should be tested)
- Diabetes mellitus
- Persons receiving immunosuppressive therapy (e.g., prolonged corticosteroid therapy [the equivalent of >15 mg/day of prednisone for one month or more], TNF-α blockers)
- Children four years of age
3. Low risk: test not indicated

**Table 3:** Target for tuberculin skin testing and who should be considered for treatment.

Tuberculin skin test reaction size, (mm induration)	Situation in which reaction is considered positive
TST≥5 mm	HIV seropositive
	Close contact with positive AFB in the sputum
	Fibrotic changes on chest radiograph consistent with prior untreated tuberculosis
	Organ transplants and other immunosuppressed patients (e.g., persons receiving the equivalent of 15 mg/day of prednisone for at least 30 days or more)
TST≥10 mm	Injection drug use
	Residents and employees in high-risk settings: nursing homes, and other long-term care facilities, hospitals
	Persons at increased risk of developing active tuberculosis: those with silicosis, diabetes mellitus, chronic renal failure, leukemia, lymphoma, cancer of the head, neck or lung, weight loss of more than 10% of ideal body weight, gastrectomy, jejunoileal bypass
	Children younger than four years of age
TST≥15 mm	Persons with no risk factors for tuberculosis
Contraindications for treatment	Any clinical, radiological, or bacteriological evidence of TB disease

Normal healthy individuals with LTBI have an annual risk of 0.1% (1 per 1000) of developing active TB.^32^ Depending on the individual's clinical condition, the annual risk of disease, if infected, can range from more than 10% for HIV patients to 1 to 2% for patients on hemodialysis.^33^–^35^

High-risk individuals are those whose risk for reactivation is at least six times higher than for normal, healthy individuals.^33^,^36^–^44^ Moderate-risk individuals are those whose risk for reactivation is 3 to 6 times higher than for normal, healthy individuals.^45^–^52^ Low-risk individuals are those at slightly increased risk for reactivation and whose risk is 1.5 to 3 times higher than for normal, healthy individuals.^53^–^55^ Table 4 shows the estimated risks for TB relative to healthy individuals in various conditions. Defining a positive tuberculin reaction is dependent on the size of the TST: ≥5 mm is considered positive in high-risk individuals whereas ≥10 mm is considered positive in moderate-risk individuals. For persons at low risk for TB and with high risk of exposure to NTM (for whom tuberculin testing is not generally indicated), ≥15 mm of induration is considered positive. Treatment for LTBI is contraindicated in patients with active TB disease and in patients with severe liver disease.^4^

**Table 4:** Relative risk for developing active tuberculosis by selected clinical conditions

Risk factor	Estimated risk for TB relative to persons with no known risk factor	References
High risk		
Acquired immunodeficiency syndrome (AIDS)	110-170	33, 36
Human immunodeficiency virus infection (HIV)	20-74	37, 41
Silicosis	30	40, 88
Chronic renal failure on hemodialysis	10.0-25.3	35, 38, 39, 89
Transplantation (related immunosuppressant therapy)	20-74	76-79
Jejunoileal bypass	27-63	90, 91
Carcinoma of head or neck	16	92, 93
Recent TB infection (≤2 years)	15	42, 43
Abnormal chest x-ray with apical fibronodular changes typical of healed TB (not granuloma)	6-19	44, 71, 94

Moderate risk	
Tumor necrosis factor (TNF)-α inhibitors	1.5-4	45, 46, 95
Treatment with glucocorticoids	4-9	47
Diabetes mellitus (all types)	2-3.6	48-51
Young age when infected (≤4 years)	2.2-5	52, 53, 72, 73

Low risk		
Underweight (<85% of ideal body weight); for most individuals, this is equivalent to body mass index (BMI) ≤20.	2-3	53
Cigarette smoker (1 pack/day)	2-3	54, 55
Chest x-ray with solitary granuloma	2	94, 96

## The principle indications and precautions in latent tuberculosis testing and treatment

1. The goal of testing for latent tuberculosis infection is to identify individuals who are at risk of new infection, and to identify individuals at increased risk of reactivation due to the biological factors listed in Table 1.
2. All people who are considered high risk should be tested, regardless of BCG history. A history of BCG vaccination should not be considered when deciding whether to perform TST or not. Furthermore, the effects of BCG on TST should be ignored in high-risk individuals.
3. NTM is not a clinically important cause of false-positive TST, particularly in Saudi Arabia, and should not be considered in the interpretation of TST.^9^,^25^–^28^
4. Patient age is of some importance in the decision to test for LTBI. Testing is not recommended for individuals ≥65 years unless they are at high risk for reactivation.^56^
5. It is essential to exclude active TB disease in persons with newly detected latent TB infection. Treatment for LTBI should not be initiated until active disease has been excluded. Medical examination and chest x-ray should be done to rule out tuberculosis disease for any individual with a newly identified positive test. Persons with radiographic signs or symptoms that are suggestive of active TB should undergo sputum smear microscopy and culture to rule out active TB.

5.1 If the radiograph is normal and the patient has no symptoms or physical findings consistent with TB, treatment of LTBI may be indicated.

5.2 If the radiograph is normal but the patient has a clinical presentation consistent with TB, further workup is indicated and treatment of LTBI should be delayed until active TB has been ruled out.

5.3 If the radiograph is abnormal and consistent with TB, specimens of sputum (using sputum induction if necessary) should be obtained. Starting treatment pending the culture results or waiting for culture results before starting therapy, is a clinical judgment dependent on benefit-risk considerations.

5.4 If a radiograph is abnormal but was taken more than three months prior to evaluation, a new radiograph should be performed at the time of evaluation.

5.5 If the abnormality on the chest radiograph is of questionable significance, consultation with an expert is recommended.

## Aims of and recommended regimens for LTBI treatment

The term, ‘treatment of LTBI’ has been adopted to highlight the fact that the patient is considered to be infected with live (although dormant) bacilli, which could cause active TB disease in the future, and that there is effective treatment available for this infection.

## 1. Aim of treatment

Treatment of LTBI is intended to prevent the development of active TB disease. In the past, this has been referred to as ‘chemoprophylaxis’ or ‘preventive therapy’. Latent tuberculosis infection is associated with a low burden of organisms; therefore, treatment of latent infection requires fewer drugs than active disease to decrease the risk of developing active tuberculosis without creating drug resistance. In most of the cases, use of a single antituberculosis agent is sufficient for the treatment of latent infection, but not for active disease.

## 2. Recommended regimens for treatment

- Four basic regimens are currently recommended (Table 5).
- Completion of therapy is based on the total number of doses administered, not the duration of therapy alone.
- Treatment interruptions of more than two months require another evaluation to exclude active TB before restarting therapy.

**Table 5:** Recommended drug regimens for the treatment of LTBI.

Drug	Interval and duration	Oral dosage (maximum)	Criteria for completion	Comments
Isoniazid	Daily×9 mo	Adult: 5 mg/kg (300 mg)	270 doses within 12 mo	INH daily for 9 months is the preferred regimen for all persons and the only regimen for persons with fibrotic lesions on CXR
		Child: 10-20 mg/kg (300 mg)		
	Twice-weekly by DOT×9 mo	Adult: 15 mg/kg (900 mg)	76 doses within 12 mo	Use twice-weekly regimen only if daily regimen not feasible. DOT must be used with twice-weekly dosing
		Child: 20-40 mg/kg (900 mg)		
Isoniazid	Daily×6 mo	Adult: 5 mg/kg (300 mg)	180 doses within 9 mos	Use ONLY if preferred regimen not feasible.
				Not recommended for HIV-infected persons† those with fibrotic changes on CXR, or children aged
	Twice-weekly by DOT×6 mo	Adult: 15 mg/kg (900 mg)	52 doses within 9 mo	<18 years.
				DOT must be used with twice-weekly dosing.
Rifampin	Daily×4 mo	Adult: 10 mg/kg (600 mg)	120 doses within 6 mo	Use ONLY if preferred regimen not feasible.
		[Child: 10-20 mg/kg (600 mg) see comments]		Not recommended for persons with fibrotic changes on CXR.
				Not recommended for persons aged <18 years, unless exposed to INH-resistant, RIF-susceptible TB.
				For HIV-infected persons, most protease inhibitors or non-nucleoside reverse transcriptase inhibitors should not be administered concurrently with RIF. Consult web-based updates for the latest specific recommendations.

RIF + PZA: Generally should not be offered for treatment of LTBI.

- Isoniazid alone—preferred regimen
  Daily INH for 9 months is the preferred regimen for the treatment of LTBI.^4^,^57^–^59^
  - Daily isoniazid INH for a 9 month regimen for children and adolescents (up to age 18 years).
  - Daily INH for a nine-month regimen for HIV-infected persons or persons suspected of having HIV infection.
  - Daily INH for a nine-month regimen for immunocompetent adults.
  Intermittent (twice weekly) INH for nine months has not been compared directly with daily administration but is considered to be an acceptable alternative, provided all doses of therapy are directly observed.^4^
- Six months INH alone—acceptable alternative:
  - INH may be given for six months if there are any concerns about side effects or adherence.
- Rifampin for 4 to 6—acceptable alternative
  This regimen should be reserved for those individuals who cannot tolerate INH or for persons exposed to cases with resistance to INH, but susceptible to RIF.
  - Rifampin (RIF) alone for four months is generally recommended for adults.
  - Rifampin alone for six months is generally recommended for children.^60^
  - Rifabutin (RFB) may be substituted for rifampin in the above regimens in situations where rifampin cannot be given, such as in HIV-infected persons taking protease inhibitors or non-nucleoside reverse transcriptase inhibitors.^4^
- Rifampin and pyrazinamide for two months—NOT recommended
  This regimen is not recommended due to fatal hepatotoxicity. Therefore, it should generally NOT be offered as initial therapy to persons with LTBI for either HIV-negative or HIV-infected persons.

## 3. Monitoring of treatment

- Education about adverse effects associated with the treatment of LTBI, with advice to stop treatment and promptly seek medical evaluation if serious adverse effects occur.
- Follow-up evaluation at least monthly, including careful questioning and a brief physical examination to assess for evidence of hepatitis or other adverse effects, symptoms of TB disease, and adherence to the regimen.
- All persons receiving treatment for LTBI should be monitored clinically for symptoms and signs of adverse drug reactions, beginning at the first initial visit and at least monthly thereafter.
- Routine laboratory monitoring of liver function tests (LFTs) is recommended for persons with baseline abnormalities in LFTs or other risk factors for drug-induced hepatitis. Additional testing is indicated if symptoms or signs indicative of hepatitis develop.
- Indications for baseline LFTs, including serum bilirubin and either aspartate aminotransferase (AST) or alanine aminotransferase (ALT) are as follows:
  1. Person with viral hepatitis or for whom complete hepatitis serology results are unknown
  2. HIV infection
  3. Pregnancy or less than three months postpartum
  4. History or initial evaluation indicative of hepatitis or cirrhosis
  5. Regular alcohol use
  6. Use of potential hepatotoxic medication
- Indications for monthly LFTs, including serum bilirubin and either AST or ALT, are as follows:
  - Abnormal baseline LFT
  - Chronic liver disease
  - Regular alcohol use
  - Current use of hepatotoxic drugs
- We recommend withholding INH if serum transaminase concentrations exceed three times the upper limit of the normal range when accompanied by symptoms, or five times the upper limit of the normal range in asymptomatic patients.

## Treatment of latent tuberculosis in special situations

### Pregnancy/lactation

Pregnancy has minimal influence on the pathogenesis of TB and there is no evidence to suggest a greater risk of progression of LTBI to active disease during pregnancy.^4^ The treatment of LTBI during pregnancy remains controversial. However, under ordinary circumstances, physicians should delay treatment until two to three months after delivery.^4^ However, for pregnant women who are HIV-positive or at high risk of progression to active disease and to prevent its consequences on the mother and the fetus, initiation of therapy should not be delayed on the basis of pregnancy alone, even during the first trimester.^4^ INH can be given to pregnant women and is not toxic to the unborn child, even during the first four months of gestation.^4^,^7^,^61^–^66^ Pregnant women taking INH should receive vitamin B6. Breastfeeding is not contraindicated when the mother is being treated for LTBI.^4^

### Treatment of HIV-infected persons

Recommendations for HIV-infected adults are in general similar those for HIV-negative adults, although the quality of evidence and strengths of the recommendations vary.^4^,^67^–^70^ If isoniazid is chosen for treatment of LTBI in persons with HIV infection, nine months of therapy is recommended rather than six months. In addition, rifampin should generally be avoided in persons who are taking protease inhibitors (PIs) or NNRTIs.^4^,^7^ However, the management of persons co-infected with HIV and LTBI can be highly complex. Our recommendation is that management should be attempted in consultation with physicians who are experts in the treatment of TB and HIV.

### Persons with fibrotic lesions/suspected disease

Patients who have a chest radiograph demonstrating upper lobe fibronodular changes or scarring compatible with previous TB and a positive TST (>5 mm) without evidence of active disease and no history of treatment for TB, are considered at high risk of reactivation and should receive treatment.^4^,^7^,^44^,^71^ Persons with evidence suggestive of healed, primary TB (i.e., calcified solitary pulmonary nodules, calcified hilar lymph nodes, and apical pleural capping) have only slightly increased risk for TB (about double that of the healthy). Their risk for TB and need for treatment of LTBI should be determined by considering other risk factors.^4^

### Renal failure

Patients with chronic renal failure are at a high risk of reactivation (relative risk, 10.0-25.3) and all patients with positive reaction are recommended for therapy.^38^,^39^ However, anergic reaction is common among patients requiring hemodialysis, even in patients resident in endemic areas, so that TST may not be very sensitive to detect LTBI. In the absence of randomized trials that are specific for this population, nine months of isonizid (INH) is recommended.^4^

### Children and adolescents

Infants and young children (i.e., those younger than five years of age) with LTBI are at high risk of progression to active TB. If untreated, they have up to 40% likelihood of developing active TB.^4^,^52^,^72^,^73^ In particular, young children in contact with adults with active TB (i.e., household contacts) are at high risk for developing TB disease and should therefore be targeted for LTBI therapy (after ruling out active TB). The risk of progression decreases gradually through childhood. Infants and young children are more likely than older children and adults to develop life-threatening forms of TB, especially meningeal and disseminated disease. INH therapy for LTBI is more effective for children than adults, with risk reduction of 70-90%. Infants, children, and adolescents generally tolerate INH better than adults.^4^,^72^,^73^

The only recommended regimen for the treatment of LTBI in HIV-negative children is a nine-month course of INH as self-administered daily therapy or by DOT twice weekly.^4^ Routine administration of pyridoxine is not recommended for children taking INH, but should be given to (1) breastfeeding infants, (2) children and adolescents with diets likely to be deficient in pyridoxine, and (3) children who experience paresthesias while taking INH. RIF alone has been used for the treatment of LTBI in infants, children, and adolescents when INH could not be tolerated, or when the child has had contact with a patient infected with an INH-resistant but RIF-susceptible organism. If RIF alone is used, then a six-month treatment regimen is recommended.^4^,^72^,^73^

### Liver disease

Patients with chronic liver disease with LTBI pose a special problem, as all available regimens are potentially hepatotoxic. Four months of RIF may be safer although the efficacy of such a regimen has not been established.^74^,^75^ However, frequent clinical and laboratory monitoring for drug side effects is prudent.

### Solid organ transplantation

Solid organ transplantation is associated with a very high risk of LTBI reactivation to active TB. The incidence of infection among such patients is estimated to be 20-74 times that for the general population.^76^–^79^ They should be treated for LTBI if their TSTs are positive. Safety of INH remains a concern, especially in liver transplant recipients, but is generally safe as long as pretreatment liver transaminase levels are normal. Common strategies include INH for nine months or RIF for four months. Patients should be treated during the pretransplant period, if possible.

### Contacts of patients with drug-susceptible tuberculosis index case

Persons who are contacts of patients with drug-susceptible TB and who have positive tuberculin skin-test reactions should be treated with one of the recommended regimens listed above.^4^ In addition, some tuberculin-negative close contacts who are at high risk to develop severe active disease (e.g., children younger than five years of age) should be treated and another skin test performed a few weeks after contact has ended. If the repeat skin test is negative, the treatment should be discontinued.^31^ Immunosuppressed persons, including those with HIV infection, who are close contacts of persons with active TB, should also receive treatment, even if repeat skin testing remains negative.^4^,^31^

### Contacts of index cases with isoniazid-resistant tuberculosis

For TST-positive contacts of index or source cases with INH-resistant TB, we recommend treatment with four months of RIF; this appeared to be effective for tuberculin converters in two outbreaks of INH-resistant TB.^80^,^81^ In situations in which RIF cannot be used, rifabutin can be substituted.

### Contacts of patients with a multidrug-resistant tuberculosis index case

Treatment of LTBI after possible exposure to multidrug-resistant TB has not been evaluated in a randomized trial. For those who are at high risk of progression to active TB, preventive therapy with two drugs to which the organism is expected to be susceptible is recommended. Ideally, the selection of drugs should be guided by in vitro susceptibility test results from the isolate to which the patient was exposed and is presumed to be infected.

For persons who are likely to be infected with MDR-TB and are at high risk of developing TB, treatment should be with at least two active agents. Pyrazinamide (PZA) and ethambutol (EMB) or PZA and fluoroquinolones (FQN) have been recommended for 6 to 12 months.^82^ However, the latter regimen is very poorly tolerated;^83^ hence, we recommend an FQN (levaquin or moxifloxacin) and EMB as the preferred regimen for adults. PZA and EMB are recommended for 9 to 12 months for children because FQN use is contraindicated in pediatric patients.^4^,^31^ All persons with suspected latent MDR-TB infection should be followed for at least two years, regardless of the treatment regimen. When FQN and EMB cannot be used, experts recommend using a combination of two other drugs to which the infecting organism is likely to be susceptible.

## Use of interferon release assays

A major advance in recent times has been the development of T-cell-based interferon-gamma release assays (IGRAs).^84^,^85^ IGRAs are in vitro tests that are based on interferon-gamma (IFN-Y) release after stimulation by antigens (such as early secreted antigenic target 6 [ESAT-6] and culture filtrate protein 10 [CFP-10]) which are more specific to MTB than the purified protein derivative (PPD). Two IGRAs are currently available as commercial kits that are US Food and Drug Administration (FDA)-approved, and marked for use in Europe: the QuantiFERON-TB Gold® In-Tube (QFT) assay (Cellestis Ltd., Carnegie, Australia) and the T-SPOT.TB® assay (Oxford Immunotec, Abingdon, UK). Of the two commercial tests, the QFT assay is simple and easy to use because of its ELISA format, and less expensive.

There are many potential advantages of these new tests. They are standardized and quality-controlled laboratory tests that provide results in a single patient visit. They do not have a ‘booster’ effect, and can be repeated without the need for two-step testing. They have excellent specificity and are unaffected by BCG and NTM (estimated specificity >98%).^84^,^85^ The high specificity of IGRAs is likely to be useful in BCG-vaccinated individuals, particularly in countries where TST specificity is compromised by BCG vaccination after infancy or by multiple BCG vaccinations.

Sensitivity of IGRAs and TST is not consistent across tests and populations, but IGRAs appear to be at least as sensitive as the TST (estimated with active TB as the surrogate reference standard).^84^,^85^ However, IGRAs should not be used to diagnose active TB because they cannot distinguish between LTBI and active disease.

IGRAs have some disadvantages, including higher material cost, the need for an equipped laboratory, and a requirement to draw peripheral blood. Evidence is still limited on the prognostic (predictive) value of these tests, and their added value in TB diagnosis and control. Furthermore, the interpretation of IGRA conversions and reversions is unclear, and evidence is rather limited in children and immunocompromised populations. Many studies on IGRA are ongoing in this area to properly define the role of IGRA in day-to-day clinical practice.^86^

### Use of IGRA for the diagnosis of latent TB infection

The use of IGRAs is steadily increasing in low or intermediate incidence countries. More than a dozen countries (e.g., USA, UK, Canada, Germany, France, Switzerland, Japan, Netherlands) now have at least one guideline or statement on the use of IGRAs. There is considerable diversity in how various countries currently recommend and use IGRAs. For example, the CDC guidelines for the USA recommend using either TST or IGRA for LTBI testing. In contrast, many countries (e.g., UK, Canada, Spain, Italy) recommend a two-step approach where TST is done first, followed by IGRA. Globally, the two-step approach seems to be the most popular and possibly a very cost-effective strategy. However, because of the potential boosting effect of TST on IGRA results,^87^ it may be best to collect blood for IGRA at the time the TST is read (i.e., within three days of placing PPD).

### Recommendations

1. IGRAs should not be used to diagnose active TB in adults. In children, they may be used as an adjunct test, in combination with TST, chest x-ray, and microbiological investigations. A negative IGRA alone should not be used to rule out active TB.
2. In BCG-vaccinated individuals (adults and children), IGRAs may be used to confirm a positive TST result (i.e., to check if the TST result is a false positive). If a positive TST result is confirmed by a positive IGRA result, LTBI treatment should be initiated after ruling out active TB.
3. In immunocompromised patients, if a false-negative TST result is suspected, IGRAs may be used to rule out LTBI.

### Conclusions

Saudi Arabia is a country of medium prevalence for TB infection. The TST remains a useful tool in the identification of subjects with latent TB infection. NTM infection and BCG vaccination (routinely given in infancy) do not interfere with the interpretation of the test in adolescents and adults. Patients at high risk of developing active TB should be treated with the standard regimens advocated in this statement after due care is taken in excluding active TB disease.
